# Icariin promotes osteogenic differentiation through the mmu_circ_0000349/mmu-miR-138-5p/Jumonji domain-containing protein-3 axis

**DOI:** 10.1016/j.heliyon.2023.e21885

**Published:** 2023-11-06

**Authors:** Liang Ai, Liudan Chen, Yangu Tao, Haibin Wang, Weimin Yi

**Affiliations:** a Department of TCM, Sun Yat-Sen Memorial Hospital, Sun Yat-Sen University, Guangzhou, Guangdong, 510120, China; b Department of TCM and Acupuncture, Sun Yat-Sen Memorial Hospital, Sun Yat-Sen University, Guangzhou, 510120, China; c Department of Orthopaedics, The First Affiliated Hospital of Guangzhou University of Chinese Medicine, Guangzhou, 510405, China

**Keywords:** JMJD3, circ_0000349, miR-138-5p, Osteogenic differentiation, Icariin

## Abstract

Circular RNAs (circRNAs) regulate Jumonji domain-containing protein-3 (JMJD3) by sponging with microRNAs (miRNAs). This study aimed to investigate the role of icariin on specific circRNA/miRNA/JMJD3 axis in osteogenic differentiation of MC3T3-E1 cells. CircRNA sequencing was performed on the MC3T3-E1 cells induced by osteogenic differentiation medium for 1 d (negative control (NC) group) and 14 d (osteogenesis group). And mmu_circ_0000349 was verified using Sanger sequencing, ribonuclease R degradation, and actinomycin D assay. The function of mmu_circ_0000349 was validated by detecting the expressions of osteogenic differentiation markers, alkaline phosphatase (ALP), and runt-related transcription (RUNX2), *via* real-time quantitative PCR (qPCR) and Western blotting or ALP and alizarin red staining assay. Dual luciferase reporter gene assay confirmed the relationship between mmu_circ_0000349 and mmu-miR-138-5p (or mmu-miR-138-5p and JMJD3). Meanwhile, the JMJD3 binding to mmu_circ_0000349 was screened using an RNA pull-down assay. qPCR and Western blotting confirmed the effect of icariin on the mmu_circ_0000349/mmu-miR-138-5p/JMJD3 axis and osteogenic differentiation. As MC3T3-E1 osteogenic differentiation progressed, the JMJD3 expression level increased. A total of 361 circRNAs exhibited differences between the NC and osteogenesis groups. After validation, mmu_circ_0000349 was further analyzed as it exhibited the largest expression. And mmu_circ_0000349 was identified as a stable circular structure. Overexpression of mmu_circ_0000349 increased the expression levels of ALP and RUNX2, enhanced ALP activity, and increased the number of mineralized nodules; contrarily, inhibition of mmu_circ_0000349 exerted opposite effects. The data also confirmed that mmu_circ_0000349 regulated JMJD3 by sponging with mmu-miR-138-5p. With the increase in icariin concentration and time for treatment, the expression levels of mmu_circ_0000349, JMJD3, ALP, and RUNX2 also increased, whereas that of mmu-miR-138-5p decreased. In conclusion, Icariin promoted osteogenic differentiation by regulating the mmu_circ_0000349/mmu-miR-138-5p/JMJD3 pathway. Therefore, this provides a theoretical basis for the treatment of diseases related to osteogenic differentiation.

## 1 Introduction

Imbalance between osteoclastic bone formation and bone resorption is observed in different diseases, including osteoporosis, bone defects, osteoarthritis, osteogenesis imperfecta, periodontitis, and osteonecrosis of the femoral head [[1], [2], [3], [4], [5]]. This suggests that bone quality depends on the balance between the two. Osteoblasts are bone-forming cells that undergo proliferation, differentiation, and mineralization to become mature osteoblasts [6,7]. A successful osteogenic differentiation is important for bone formation [8]. In the progression of osteogenic differentiation, runt-related transcription factor 2 (RUNX2) is activated, which promotes the expressions of osteogenic genes such as ALP, osteonectin, and osteopontin [5]. In many previous studies, mouse pre-osteoblast MC3T3-E1 cells have been used for the mechanism research of osteogenic differentiation [8,9]. In this study, the same cells were used.

JMJD3 is a histone demethylase, also known as KDM6B. It regulates chromatin modifiers to induce the transcription of genes, such as cancer-related, inflammation-related, and developmental genes, by demethylating the repressive H3K27me3 markers on their promoters and gene bodies [10]. This explains why JMJD3 is involved in different types of diseases. Previous studies have reported that JMJD3 plays a vital role in the osteogenic differentiation of periodontal ligament cells as well as dental and human mesenchymal stem cells [[11], [12], [13], [14]]. In periodontal ligament cells, the silence of JMJD3 reduces osteogenic differentiation by inhibiting the expressions of RUNX2, osteonectin, and osterix, genes involved in bone formation [14]. JMJD3 knockdown reduces ALP activity and the formation of mineralized nodules, and this phenomenon is restored by the overexpression of JMJD3 in dental mesenchymal stem cells [11]. MicroRNA 146a inhibits JMJD3 expression to block the RUNX2 expression and differentiation of human mesenchymal stem cells [13].

Circular RNAs (circRNAs) have gained increasing attention in recent years owing to their stability, high evolutionary conservation, and abundant expression in cells and tissues [15]. CircRNAs play an influential role by acting as sponge of microRNAs, gene transcription, coding functions, and protein binding in different biological progress, such as proliferation, migration, and osteogenic differentiation [16]. High-throughput RNA sequencing and the use of bioinformatics methods accelerate the discovery of circRNAs. For example, circRNA HGF acts as a sponge of microRNA-25-3p to regulate smad 7 and participates in the proliferation and osteogenic differentiation of bone marrow mesenchymal stem cells [17]. Furthermore, circ_0018168 overexpression reduces fibroblast proliferation and osteogenic differentiation in ankylosing spondylitis by promoting DKK1 expression *via* miR-330-3p adsorption [18]. In addition, previous studies have demonstrated that JMJD3 can be regulated by microRNAs. Exosomal microRNA-138-5p derived from breast cancer cells suppresses M1 polarization but promotes M2 polarization by inhibiting the expression of JMJD3 [19]. Exosomal microRNA-27b produced by mesenchymal stem cells targets JMJD3 to inhibit sepsis development by inactivating the NF-κB pathway [20]. Furthermore, microRNA-939 targets JMJD3, and exerts an anti-hepatitis B virus effect [21]. Whether there are circRNAs that regulate JMJD3 by targeting microRNAs in osteogenic differentiation still needs further investigation.

Icariin, a type of flavonoid glycoside, is used to clinically treat inflammation, cerebrovascular diseases, and osteoporosis [22,23]. Previous studies have reported that icariin plays a role in osteogenic differentiation [22,24]. Furthermore, icariin promotes microRNA-335-5p expression to induce osteogenic differentiation of bone marrow stem cells [22]. It also promotes osteonecrosis of the femoral head by decreasing microRNA-23a-3p expression and regulating the BMP-2/Smad5/RUNX2 and WNT/β-catenin pathways [24]. However, the relationship between icariin and JMJD3 remains unknown.

Based on the above researches, this study aimed to investigate whether icariin regulates JMJD3 to participate in osteogenic differentiation by regulating the circRNA/microRNA/JMJD3 axis.

## 2 Materials and methods

### 2.1 Cell culture

Mouse pre-osteoblast MC3T3-E1 cells were purchased from CellCook (#CC9024, Guangzhou, China) and cultured in alpha MEM (#CM2003, CellCook) supplemented with 10 % fetal bovine serum. The cells were amplified in 5 % CO_2_ humidified incubator at 37 °C.

### 2.2 Cell treatment

For osteogenic differentiation, MC3T3-E1 cells were cultured in the OriCell® mouse MC3T3-E1 cells osteogenic differentiation kit (#MUXMT-90021, Cyagen, Guangzhou, China) for 1, 3, 7, and 14 d. Then MC3T3-E1 cells were treated with 0, 0.1, 1, 10, 100 μM icariin (Purity: 99.06 %; #I811797-20, Macklin, Shanghai, China) for 48 h or 10 μM icariin for 0, 12, 24, 48 h. Icariin was dissolved in dimethyl sulfoxide (DMSO; cat no. D2650-100 ML, Sigma-Aldrich). An equal volume of DMSO was used as control. To overexpress or inhibit mmu_circ_0000349, Lipofectamine 3000 (#L3000001, Thermo Fisher, Guangzhou, China) was used to transfected pcDNA3.1-mmu_circ_0000349 (pcDNA3.1-circRNA) or siRNAs (si-circRNA) into MC3T3-E1 cells. After 48 h, the cells were collected for further analysis. The pcDNA3.1 negative control (pcDNA3.1-NC) and negative control of siRNAs (si-NC) were the control of pcDNA3.1-circRNA and si-circRNA, respectively. Meanwhile, MC3T3-E1 cells were treated with 1 μM actinomycin D (MCE, NJ, USA; cat. no. HY-17559) for 0, 1, 3, 6, and 12 h.

### 2.3 Real-time quantitative PCR (qPCR) and Sanger sequencing

In this study, RNA was separated from the cells using the TriQuick Reagent Total RNA Isolation Kit (#R1100, SolarBio, Beijing, China), which was then reversed into cDNA using HiScript III RT SuperMix for qPCR (+gDNA wiper) (#R323-01, Vazyme, Guangzhou, China). PCR was performed using ChamQ Universal SYBR qPCR Master Mix (#Q711-02, Vazyme). The primer sequences are presented in Table 1. The relative expression levels were calculated using the 2^−△△Ct^ method. For mRNA and circRNA, GAPDH was the internal control, whereas for microRNA, it was U6. The PCR products of mmu_circ_0000349, mmu_circ_0000679, and mmu_circ_0000061 were used for Sanger sequencing (Sangon, Guangzhou, China) to confirm the circular structure [25].

**Table 1:** The primer sequences used in this study.

Primer Name	Sequence (5′-3′)	Product length（bp）
JMJD3-F	CCCAGGCCCTGTGAGTAAAG	156
JMJD3-R	TTTGCCAGCCCATCAGGTAG	
ALP-F	GGGCAATGAGGTCACATCCA	85
ALP-R	GTGGTTCACCCGAGTGGTAG	
Runx2-F	GGCCACTTACCACAGAGCTA	122
Runx2-R	GCCCTAAATCACTGAGGCGA	
mmu_circ_0000349-F	GTGGAATGCTCCTACTGTCACT	220
mmu_circ_0000349-R	TCCACCACACATGGTTGCTAA	
mmu_circ_0000679-F	CATCGTGGCTCAACAGGACTT	155
mmu_circ_0000679-R	TCTAAACACACACTGTTCACCGA	
mmu_circ_0000061-F	TCTAGGAAAGGGAGCGACCC	126
mmu_circ_0000061-R	GCTTCTTCCTTGTGGCAACG	
GAPDH-F	AGGTCGGTGTGAACGGATTTG	123
GAPDH-R	TGTAGACCATGTAGTTGAGGTCA	
miR-138-5p-RT	GTCGTATCCAGTGCAGGGTCCGAGGTATTCGCACTGGATACGACCGGCCT	101
miR-138-5p-F	AGCTGGTGTTGTGAATCAGG	
Universe-R	GTGCAGGGTCCGAGGT	
M-Dtnb-F	AGCTCCATTCAGGCGCA	95
M-Dtnb-R	CAGAAAGACGGACAGGAATGTG	
U6–F	CTCGCTTCGGCAGCACA	94
U6-R	AACGCTTCACGAATTTGCGT	

### 2.4 Ribonuclease R (RNase R) treatment

The extracted RNAs were processed with 1U RNase R (Servicebio, Wuhan, China; cat. no. G3462) for 2 and 10 min. And mmu_circ_0000349 and Dtnb mRNA expressions were verified by qPCR.

### 2.5 Nuclear and cytoplasmic RNA extraction

Cytoplasm and nuclear RNA were extracted with a Cytoplasmic and Nuclear RNA Purification Kit (Norgen Biotek, Canada; cat. no. NGB-21000) in line with the manufacturer's instruction.

### 2.6 Western blotting

Total protein was isolated using RIPA buffer. The total protein concentration was determined using the BCA method. Then, 25-μg total protein was separated *via* SDS-PAGE and transferred to the PVDF membrane. The membrane was incubated in primary antibodies followed by secondary antibodies. Finally, the protein bands were observed *via* ECL. The primary antibodies used were nati-JMJD3 (#A17382, ABclonal, Wuhan, China), anti-ALP (#11187-1-AP, Proteintech, Wuhan, China), anti-RUNX2 (#20700-1-AP, Proteintech), and anti-GAPDH (#60004-1-Ig, Proteintech), with GAPDH as the internal control. On the other hand, the secondary antibodies used were Goat Anti-rabbit IgG, peroxidase conjugated, H + L (#BL003A, Biosharp, Guangzhou, China) and Goat Anti-mouse IgG, peroxidase conjugated, H + L (#BL001A, Biosharp, Guangzhou, China).

### 2.7 ALP and alizarin red staining assay

MC3T3-E1 cells were treated under the indicated condition. Then, ALP and alizarin red staining assay were performed using the Alkaline Phophatase Staining Kit (Modified Gomori Ca–CoS Method) (#G1481, SolarBio) according to the manufacturer's protocols and alizarin red staining solution (#G1038-100 ml, Servicebio, Wuhan, China).

### 2.8 CircRNA sequencing and bioinformatics analysis

MC3T3-E1 cells cultured in osteogenic differentiation medium for 1 and 14 d were collected for circRNA sequencing. The experiment protocol was performed as described previously [26]. circRNAs were considered as differentially expressed if the fold-change cut-off value was ≥1.5 and *P* < 0.05. The target mRNAs of differentially expressed circRNAs were used for the Gene Ontology (GO, http://geneontology.org/) and Kyoto Encyclopedia of Genes and Genomes (KEGG, http://www.kegg.jp/) analyses as described previously [26]. Furthermore, these differentially expressed circRNAs were used for the circRNA/microRNA/mRNA network. As JMJD3 was the focus of our study, a circRNA/microRNA/JMJD3 network was also constructed using differentially expressed circRNAs.

### 2.9 Dual luciferase reporter gene assay

The binding sites between mmu_circ_0000349 and mmu-miR-138-5p (mmu-miR-138-5p and JMJD3) were predicted using TargetScan. Then, the wild-type (including mmu_circ_0000349 wild type 1 (circRNA 3′UTR wt1), mmu_circ_0000349 wild type 2 (circRNA 3′UTR wt2), JMJD3 3′UTR wt1, and JMJD3 3′UTR wt2) and mutant-type (including mmu_circ_0000349 mutant type 1 (circRNA 3′UTR mut1), mmu_circ_0000349 mutant type 2 (circRNA 3′UTR mut2), JMJD3 3′UTR mut1, and JMJD3 3′UTR mut2) vectors were synthesized in Cencefe (Jiangsu, China). The mmu-miR-138-5p mimics (miR-138-5p mimics) and mimics negative control (mimics NC) were provided also by Cencefe. The indicated vector and mimics were co-transfected into 293T for 48 h; then, cells were collected for luciferase activity detection using the TransDetect® Double-Luciferase Reporter Assay Kit (#FR201-01, Transgen, Beijing, China) according to the manufacturer's protocols.

### 2.10 RNA pull-down assay

Negative probe and miR-138-5p probe labeled with biotin were synthesized by Genecefe Biotechnology Co., Ltd (Jiangsu, China). After mmu_circ_0000349 overexpression, the interaction between mmu_circ_0000349 and JMJD3 in MC3T3-E1 cells was confirmed by RNA pull-down kit (IEMed, cat. no. K303) based on the manufacturer's instruction.

### 2.11 Statistical analysis

All experiments were repeated three times independently.

Data was expressed as means ± standard deviation (SD). Comparisons between the two groups were performed using Student's *t*-test; on the other hand, analysis of variance was employed for three or more groups. *P* < 0.05 was considered to indicate statistical significance. Aside from circRNA sequencing, other data was analyzed using GraphPad Prism 7.0 and SPSS 10.1.

## 3 Results

### 3.1 The expression of JMJD3 was promoted in the progression of osteogenic differentiation of MC3T3-E1 cells

**Fig. 1:**
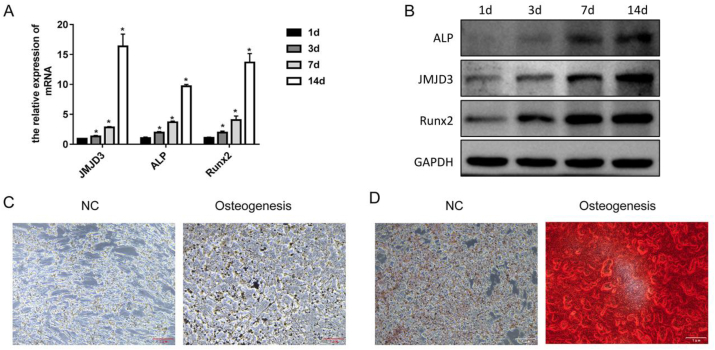
**The expression levels of JMJD3 were increased in the progress of osteogenic differentiation of MC3T3-E1 cells**. MC3T3-E1 cells were cultured in osteogenic differentiation medium for an indicated time; then, the cells were collected for qPCR (A) and Western blotting to detect the expression levels of ALP, JMJD3, and RUNX2. GAPDH was used as the internal control. (C) After the induction of osteogenic differentiation for 1 d[NC] group or 14 d (osteogenesis group), the MC3T3-E1 cells were collected for the detection of ALP activity. (C) After the induction of osteogenic differentiation for 1 d (NC group) or 14 d (osteogenesis group), the MC3T3-E1 cells were collected for alizarin red staining.

MC3T3-E1 cells were cultured in osteogenic differentiation medium for 1, 3, 7, and 14 d, and the expressions of osteogenic differentiation markers, ALP, and RUNX2, were confirmed *via* qPCR, and Western blotting. Compared with the 1-d culture, the mRNA levels of ALP and RUNX2 significantly increased in the 3, 7, and 14-d culture; especially in the 14-d culture, 10-, and 15-fold increases for ALP and RUNX2, respectively, were observed (Fig. 1A). The protein levels of ALP and RUNX2 also increased in the 3-, 7-, and 14-d culture compared with the 1-d culture (Fig. 1B). There was a significant increase in ALP activity and number of mineralized nodules in the osteogenesis group compared with the NC group (induction for 1 d) (Fig. 1C and D). These results indicated that the induction of osteogenic differentiation was successful. In the progression of osteogenic differentiation, the expression level of JMJD3 increased after induction for 3, 7, and 14 d compared with 1 d.

### 3.2 circRNA sequencing was used to analyze the differences in the expression levels of circRNAs between the NC and osteogenesis groups

**Fig. 2:**
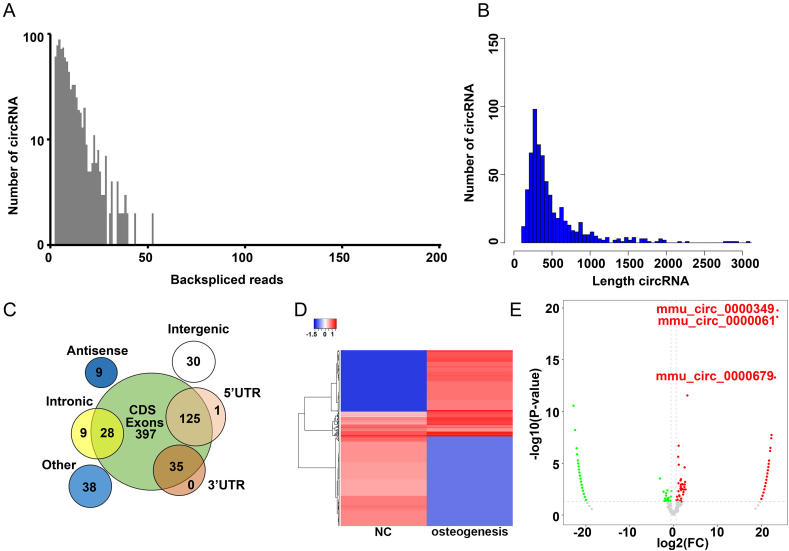
**circRNA sequencing was used to analyze the difference in the expression levels of circRNA between the NC (MC3T3-E1 cells were induced for osteogenic differentiation for 1d) and osteogenesis (MC3T3-E1 cells were induced for osteogenic differentiation for 14 d) groups.** (A) Frequency distribution of circRNA expression. X-axis: range of total backspliced reads of a circRNA in the NC and osteogenesis groups; Y-axis: frequency of circRNAs possessing a specific number of backspliced reads. (B) Frequency distribution of the circRNA length. X-axis: range of circRNA length in the NC and osteogenesis groups. Y-axis: frequency of circRNAs with specific length. (C) Genomic origin of circRNAs detected in our study. (D) Heatmap of all differentially expressed circRNAs between the NC and osteogenesis groups. (E) Volcano plot of circRNA expression. X-axis: log 2 ratio of circRNA expression level between the NC and osteogenesis groups; Y-axis: the FDR value (-log 10 transformed) of circRNA.

To investigate the changes in the expression levels of circRNAs between the NC and osteogenesis groups, MC3T3-E1 cells cultured in osteogenic differentiation medium for 1 and 14 d were collected for circRNA sequencing. A total of 671 circRNAs were detected in the NC and osteogenesis groups. The backspliced reads of circRNAs in the NC and osteogenesis groups were mainly distributed between 0 and 50 (Fig. 2A), and the length of these circRNAs was mainly 50–1000 bp (Fig. 2B). Most of the circRNAs originated from CDS exons, possessed 87.48 % (Fig. 2C). Furthermore, a total of 361 circRNAs had a fold-change cut-off value of ≥1.5 and *P* value of <0.05 (Fig. 2D and E), thus meeting the criteria for differentially expressed circRNAs.

### 3.3 The expressions of mmu_circ_0000349, mmu_circ_0000679, and mmu_circ_0000061 were promoted in osteogenic cells

**Fig. 3:**
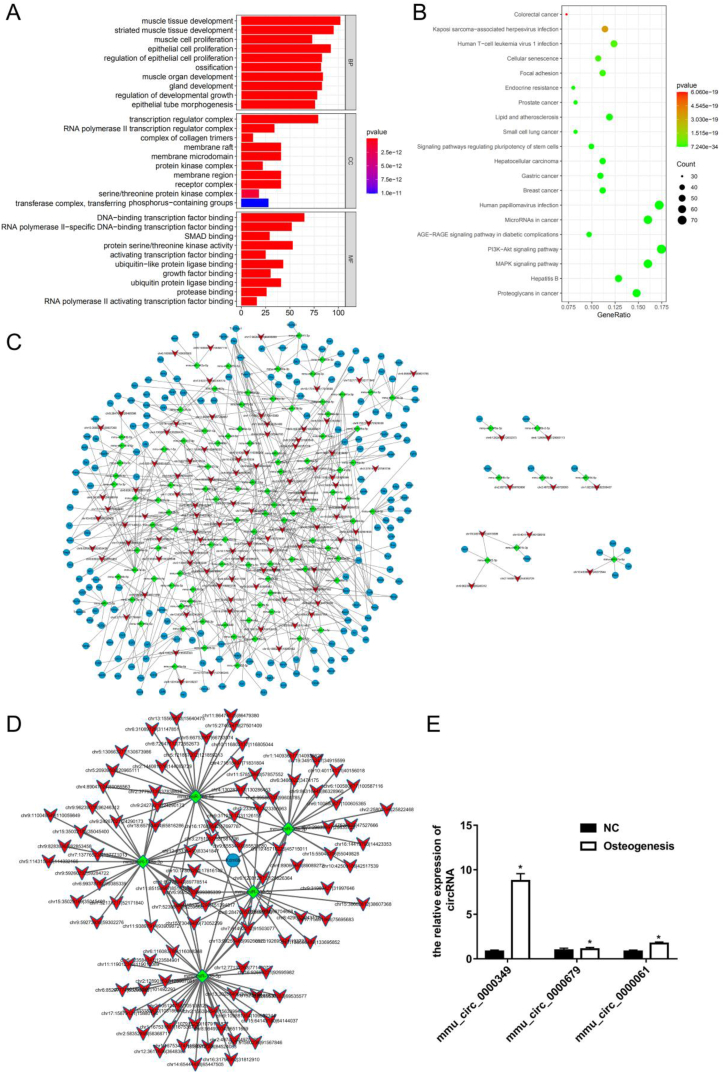
**The expression levels of mmu_circ_0000349, mmu_circ_0000679, and mmu_circ_0000061 were promoted in osteogenic cells.** (A) Differentially expressed circRNAs were used for the GO analysis; the top 10 terms of biological process (BP), molecular function (MF), and cellular component (CC) are presented. (B) Differentially expressed circRNAs were used for the KEGG analysis; the top 20 terms are presented. (C) A circRNA/microRNA/mRNA network was constructed using differentially expressed circRNAs based on the following criterion: two or more binding sites between circRNAs and microRNAs (or microRNAs and mRNAs). (D) A circRNA/microRNA/JMJD3 network was constructed according to the following criterion: two or more binding sites between circRNAs and microRNAs (or microRNAs and JMJD3). (E) The expression levels of mmu_circ_0000349, mmu_circ_0000679, and mmu_circ_0000061 were detected *via* qPCR in the NC (MC3T3-E1 cells were induced for osteogenic differentiation for 1 d) and osteogenesis (MC3T3-E1 cells were induced for osteogenic differentiation for 14 d) groups.

Differentially expressed circRNAs were used for the GO and KEGG analyses. Fig. 3A presents the top 10 terms of biological process (BP), molecular function (MF), and cellular component (CC): BP included epithelial cell proliferation, regulation of epithelial cell proliferation, ossification, etc.; CC included transcription regulator complex, complex of collagen trimers, receptor complex, etc.; and MF included Smad binding, protein serine/threonine kinase activity, activating transcription factor binding, etc. (Fig. 3A). The top 20 KEGG terms included the PI3K-AKT and MAPK signaling pathways (Fig. 3B). A circRNA/microRNA/mRNA network was constructed using differentially expressed circRNAs according to the following criterion: two or more binding sites between circRNAs and microRNAs (or microRNAs and mRNAs) (Fig. 3C). To investigate the potential circRNAs and microRNAs that regulate JMJD3, a circRNA/microRNA/JMJD3 network was constructed (Fig. 3D). A total of 99 circRNAs and 5 microRNAs were found to potentially regulate JMJD3 (Fig. 3D). Subsequently, four circRNAs were selected for qPCR validation using the following criteria: (1) the same expression trend with JMJD3, (2) a circBase ID, and (3) two binding sites between microRNAs and JMJD3. The results presented that the expression levels of mmu_circ_0000349, mmu_circ_0000679, and mmu_circ_0000061 increased in the osteogenesis group (MC3T3-E1 cells were induced for osteogenic differentiation for 14 d) than in the NC group (MC3T3-E1 cells were induced for osteogenic differentiation for 1 d); however, the expression level of mmu_circ_0000060 could not be detected (Fig. 3E). Therefore, mmu_circ_0000349 had the largest difference in expression level between the NC and osteogenesis groups, and it was selected for further analysis.

### 3.4 Characterization of mmu_circ_0000349

**Fig. 4:**
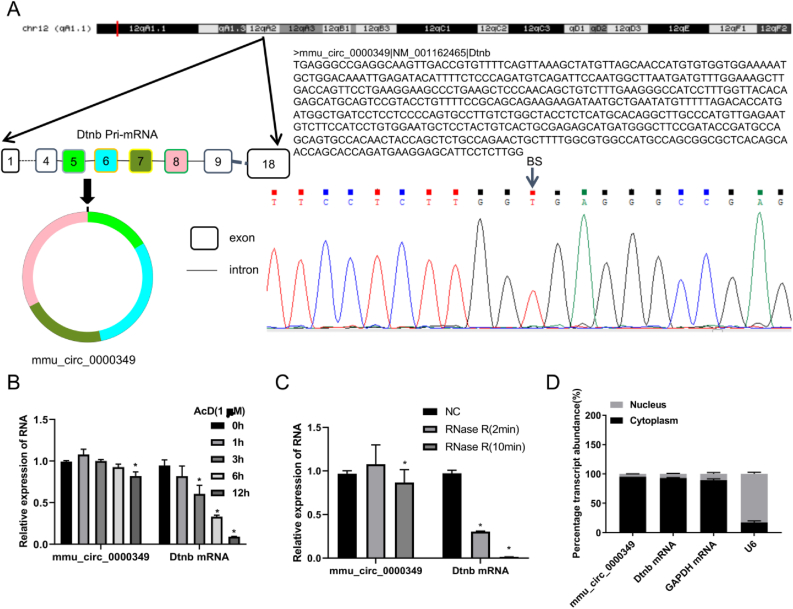
**Characterization of mmu_circ_0000349.** (A) The sketch of genomic locus of mmu_circ_0000349 in Dtnb gene, and the circular structure of mmu_circ_0000349 was verified *via* Sanger sequencing. (B) MC3T3-E1 cells were treated with 1 μM actinomycin D for 0, 1, 3, 6, and 12 h, then mmu_circ_0000349 and Dtnb mRNA expressions were analyzed by qPCR. (C) The extracted RNA was processed with 1U RNase R for 2 and 10 min, then mmu_circ_0000349 and Dtnb mRNA expressions were examined by qPCR. (D) qPCR analysis of mmu_circ_0000349 and Dtnb mRNA obtained from the nuclei and cytoplasm of MC3T3-E1 cells.

To further confirm the circular structure of mmu_circ_0000349 from Dtnb mRNA, qPCR products of mmu_circ_0000349 were used for Sanger sequencing, and the results indicated that mmu_circ_0000349 had a circular structure, and the splice junction was TTCCTCTTGGTGAGGGCCGAG (Fig. 4A). qPCR results also revealed that the expression of Dtnb in MC3T3-E1 cells was time-dependently decreased following treatment with actinomycin D, while the expression of mmu_circ_0000349 was not affected by actinomycin D (Fig. 4B). Meanwhile, we proved that the expression of Dtnb mRNA but not mmu_circ_0000349 was markedly reduced following processing with RNase R, suggesting that mmu_circ_0000349 is resistant to RNase R treatment (Fig. 4C). Then we discovered that mmu_circ_0000349 and Dtnb mRNA were mainly located in the cytoplasm (Fig. 4D). In summary, mmu_circ_0000349 has a circular structure and is relatively stable.

### 3.5 Overexpressed mmu_circ_0000349 promoted osteogenic differentiation, whereas decreased mmu_circ_0000349 inhibited osteogenic differentiation

**Fig. 5:**
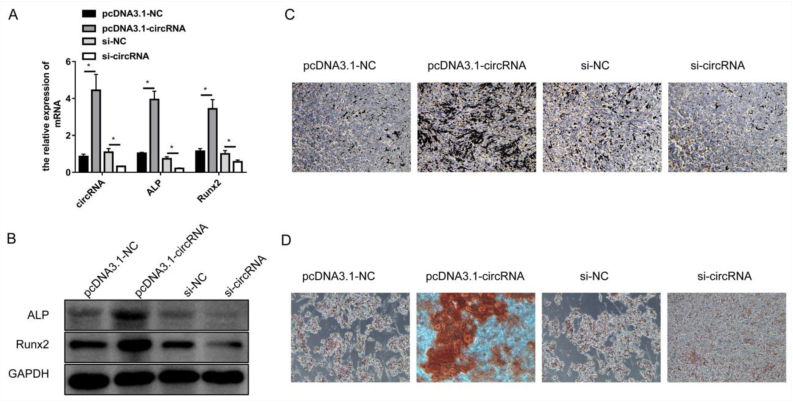
**Overexpressed mmu_circ_0000349 promoted osteogenic differentiation whereas decreased mmu_circ_0000349 inhibited osteogenic differentiation of MC3T3-E1 cells.** (A&B) MC3T3-E1 cells were transfected with pcDNA3.1-NC, pcDNA3.1-circRNA (mmu_circ_0000349), si-NC (negative control of siRNAs), and si-circRNA (siRNAs for mmu_circ_0000349) for 48 h and then cultured in osteogenic differentiation medium for another 48 h; then, the cells were collected for qPCR (A) and Western blotting (B) to detect the expression levels of mmu_circ_0000349, ALP, and RUNX2. (C) MC3T3-E1 cells were transfected with pcDNA3.1-NC, pcDNA3.1-circRNA, si-NC, and si-circRNA for 48 h and then cultured in osteogenic differentiation medium for 14 d; the cells were collected for ALP activity detection. (D) MC3T3-E1 cells were transfected with pcDNA3.1-NC, pcDNA3.1-circRNA, si-NC, and si-circRNA for 48 h and then cultured in osteogenic differentiation medium for 14 d; the cells were collected for alizarin red staining.

The role of mmu_circ_0000349 in osteogenic differentiation was investigated. The overexpression effect of pcDNA3.1-circRNA and the interference effect of si-circRNA were confirmed *via* qPCR in MC3T3-E1 cells (transfected with vector or siRNAs for 48 h and then cultured in osteogenic differentiation medium for 48 h). The results in Fig. 5A show that compared with pcDNA3.1-NC, pcDNA3.1-circRNA significantly promoted mmu_circ_0000349 expression, and compared with si-NC, si-circRNA obviously decreased mmu_circ_0000349 expression. Furthermore, the mRNA, and protein levels of osteogenic differentiation markers, ALP, and RUNX2, were stimulated by pcDNA3.1-circRNA compared with pcDNA3.1-NC, whereas their expressions were suppressed by si-circRNA compared with si-NC (Fig. 5A and B). In addition, the transfected MC3T3-E1 cells were cultured in osteogenic differentiation medium for 14 d to detect ALP activity and for 14 d to perform alizarin red staining. The results indicated that pcDNA3.1-circRNA enhanced ALP activity and osteogenic calcification compared with pcDNA3.1-NC and that si-circRNA reduced ALP activity and osteogenic calcification compared with si-NC (Fig. 5C and D).

### 3.6 mmu_circ_0000349 regulated JMJD3 expression by sponging with mmu-miR-138-5p in the progression of osteogenic differentiation of MC3T3-E1 cells

**Fig. 6:**
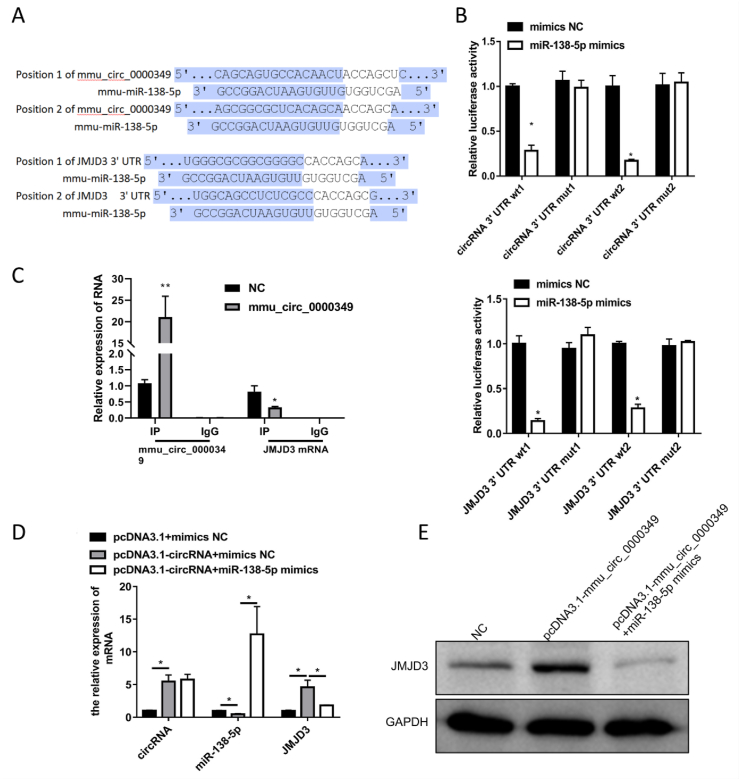
**mmu_circ_0000349 regulated JMJD3 expression by sponging with mmu-miR-138-5p in the progress of osteogenic differentiation of MC3T3-E1 cells.** (A) The binding sites between mmu_circ_0000349 and mmu-miR-138-5p (mmu-miR-138-5p and JMJD3) were predicted using TargetScan. (B) mmu_circ_0000349 (or JMJD3 3′UTR) wild- and mutant-type vectors were constructed and co-transfected with mmu-miR-138-5p mimics (or mimics NC) into 293T for 48 h; then, the luciferase activity of 293T cells was detected *via* dual luciferase reporter gene assay. (C) The interaction between mmu_circ_0000349 and JMJD3 was confirmed by RNA pull-down assay in MC3T3-E1 cells after mmu_circ_0000349 overexpression. (D) MC3T3-E1 cells were transfected with pcDNA3.1 + mimics NC, pcDNA3.1-circRNA (mmu_circ_0000349) + mimics NC, and pcDNA3.1-circRNA + mmu-miR-138-5p mimics for 48 h; then, the cells were collected for the qPCR detection of circRNA (mmu_circ_0000349), miR-138-5p, and JMJD3. (E) Same with (D) treated cells were collected for Western blotting of JMJD3; GAPDH was used as the internal control.

The results in Fig. 3D suggest that mmu_circ_0000349 can regulate JMJD3 by sponging with mmu-miR-138-5p in the osteogenic differentiation of MC3T3-E1 cells. To validate this hypothesis, the binding sites between mmu_circ_0000349 and mmu-miR-138-5p (mmu-miR-138-5p and JMJD3) were firstly predicted using TargetScan (Fig. 6A). Based on the prediction, mmu_circ_0000349 (or JMJD3 3′UTR) wild- and mutant-type vectors were constructed and co-transfected with mmu-miR-138-5p mimics (or mimics NC) into 293T for 48 h, followed by dual luciferase reporter gene assay. The results indicated that only mmu_circ_0000349 (or JMJD3 3′UTR) wild-type vector significantly decreased luciferase activity (Fig. 6B). And pull-down results denoted that overexpression of mmu_circ_0000349 could enhance the enrichment of JMJD3 mRNA, indicating that mmu_circ_0000349 can interact with JMJD3 (Fig. 6C). Furthermore, MC3T3-E1 cells were transfected with pcDNA3.1 + mimics NC, pcDNA3.1-circRNA + mimics NC, and pcDNA3.1-circRNA + mmu-miR-138-5p mimics for 48 h, and then cells were collected for qPCR and Western blotting. The results indicated that compared with the pcDNA3.1 + mimics NC group, pcDNA3.1-circRNA promoted the expression of mmu_circ_0000349 and JMJD3 (mRNA and protein levels) but inhibited that of mmu-miR-138-5p; on the other hand, compared with the pcDNA3.1-circRNA + mimics NC group, mmu-miR-138-5p mimics enhanced mmu-miR-138-5p expression and inhibited the JMJD3 expression but did not affect the expression levels of mmu_circ_0000349 (Fig. 6D and E).

### 3.7 Icariin promoted osteogenic differentiation of MC3T3-E1 cells through the mmu_circ_0000349/mmu-miR-138-5p/JMJD3 axis

**Fig. 7:**
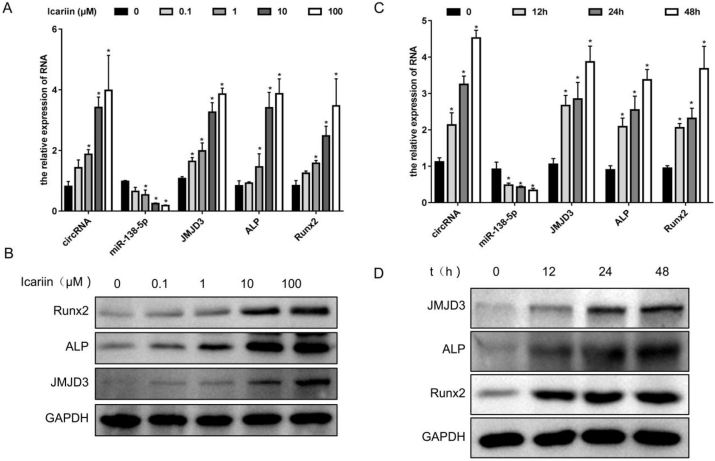
**Icariin promoted osteogenic differentiation of MC3T3-E1 cells through the mmu_circ_0000349/mmu-miR-138-5p/JMJD3 axis.** MC3T3-E1 cells were treated with icariin at different concentrations (0, 0.1, 1, 10, 100 μM) for 48 h, and mmu_circ_0000349, mmu-miR-138-5p, JMJD3, ALP, AND Runx2 expressions were analyzed by qPCR detection (A) or/and Western blotting (B). MC3T3-E1 cells were treated with 10-μM icariin at different times (0, 12, 24, 48 h), and mmu_circ_0000349, mmu-miR-138-5p, JMJD3, ALP, and Runx2 expressions were confirmed by qPCR detection (C) or/and Western blotting (D).

To confirm the effect and the potential mechanism of icariin in the progression of osteogenic differentiation of MC3T3-E1 cells, it was used at different concentrations (0, 0.1, 1, 10, 100 μM) to treat MC3T3-E1 cells at different times (0, 12, 24, 48 h). As the icariin concentration increased, the expression levels of mmu_circ_0000349, JMJD3 (mRNA and protein levels), ALP (mRNA and protein levels), and RUNX2 (mRNA and protein levels) also increased, whereas that of mmu-miR-138-5p decreased (Fig. 7A and B). In addition, the expression levels of mmu_circ_0000349, JMJD3 (mRNA and protein levels), ALP (mRNA and protein levels), and RUNX2 (mRNA and protein levels) gradually increased whereas that of mmu-miR-138-5p decreased with time (Fig. 7C and D).

## 4 Discussion

JMJD3 is a specific histone demethylase for trimethylation on histone H3 lysine 27 (H3K27me3). It promotes gene expression following the removal of H3K27me3 [27]. The expression of JMJD3 was promoted in the progression of osteogenic differentiation, which resulted in the removal of H3K27me3 at the RUNX2 promoter and then upregulation of RUNX2 expression. These events increased ALP activity and the number of mineralized nodules [27]. In this study, MC3T3-E1 cells were cultured in osteogenic differentiation medium for 1, 3, 7, and 14 d, and the expression levels of JMJD3, RUNX2, and ALP increased with time. In the 14 d culture, ALP activity, and the number of mineralized nodules increased, respectively. These results indicated that the osteogenic differentiation model of MC3T3-E1 was successfully constructed, and the expression of JMJD3 was enhanced in the progression of osteogenic differentiation.

An increasing number of circRNAs were found to be involved in osteogenic differentiation, such as circRNA-vgll3, circRNA-23525, and circRNA-CDK8 [[28], [29], [30]]. circRNA sequencing is an effective method for finding new circRNAs. Therefore, in this study, MC3T3-E1 cells cultured in osteogenic differentiation medium for 1 d (NC group) and 14 d (osteogenesis group) were collected for RNA sequencing. Bioinformatics analysis revealed that differentially expressed circRNAs may play a significant role in osteogenic differentiation *via* the PI3K-AKT and MAPK pathways. Previous study has demonstrated that circRNA BIRC6 acts as a sponge of microRNA-543 to suppress osteogenic differentiation of periodontal ligament stem cells in an inflammatory environment by inhibiting the PI3K-AKT pathway [31]. Another study also confirmed that the PI3K-AKT pathway was activated by circRNA in the induction of osteogenic differentiation of bone mesenchymal stem cells [32]. For the MAPK pathway, previous study found that circRNAs regulate this pathway in osteogenic differentiation, such as upregulated circRNA CDR1as absorbing mircoRNA-7 to upregulation of GDF5 and subsequently activating MAPK pathway in periodontal ligament stem cells. In addition, circRNA RFWD2 targets microRNA-6817-5p to activate the MAPK pathway in inducing osteogenic differentiation of human dental pulp stem cell [33]. These studies reported that the PI3K-AKT and MAPK pathways were activated in the process of osteogenic differentiation.

The present study aimed to identify the circRNAs that regulate JMJD3 in the osteogenic differentiation of MC3T3-E1. Thus, a circRNA/microRNA/JMJD3 network was constructed, and four circRNAs, namely, mmu_circ_0000060, mmu_circ_0000349, mmu_circ_0000679, and mmu_circ_0000061, were identified following RNA sequencing. mmu_circ_0000349 had the largest difference in expression level between the NC and osteogenesis groups; therefore, it was selected for further study. The results indicated that upregulated mmu_circ_0000349 promoted osteogenic differentiation of MC3T3-E1 cells, whereas silenced mmu_circ_0000349 suppressed osteogenic differentiation. To the best of our knowledge, this is the first study investigating the function of circ_0000349.

Mechanistically, mmu_circ_0000349 absorbed mmu-miR-138-5p to upregulate JMJD3. Previous studies reported that silenced miR-138-5p promoted osteogenic differentiation by targeting many mRNAs, such as forkhead box C1, RUNX2, BMPR2, and microtubule actin cross-linking factor 1 [[34], [35], [36], [37]]. Furthermore, a previous study demonstrated that JMJD3 was the target of miR-138-5p in macrophage polarization [19]. The present study also found that miR-138-5p regulated JMJD3 expression in the osteogenic differentiation of MC3T3-E1 cells.

Icariin belongs to flavonoids and is the main active ingredient of the Chinese herbal medicine Epimedium [38]. Icariin has the effect of promoting osteogenesis and inhibiting osteoclastogenesis [39]. Research showed that icariin can promote osteogenic differentiation of human bone marrow mesenchymal stem cells, improve osteogenic activity and mineralization of osteoblasts [40]. Icariin also can inhibit osteoblastic differentiation of rabbit bone marrow cells and inhibit bone resorption [41]. And icariin also could stimulate osteogenesis by miR-23a-mediated Wnt/β-catenin pathway [42]. Currently, icariin has been used to treat bone fracture and bone loss [43]. Previous studies have found that icariin regulates microRNA-335-5p and microRNA-23a-3p in the process of osteogenic differentiation [22,24]. In our study, we unexpectedly found that icariin promoted the expression of mmu_circ_0000349 and JMJD3 and inhibited miR-138-5p expression in the osteogenic differentiation of MC3T3-E1 cells.

However, the current study also has some limitations. For example, the studied role of icariin-regulated mmu_circ_0000349/mmu-miR-138-5p/JMJD3 axis in osteogenic differentiation is based only on the cellular level, and its role at the animal level is not clear. In future studies, a large number of animal experiments should be performed to verify this conclusion. The potential mechanism by which icariin regulates mmu_circ_0000349/mmu-miR-138-5p/JMJD3 axis also needs to be confirmed by further experiments. The possible downstream pathways of JMJD3 affecting osteogenic differentiation also need to be further characterized, such as PI3K-AKT and MAPK pathways. It was reported that JMJD3 could synergize with RUNX2 could promote osteoblast differentiation [44]. In future studies, we will also further explore the effect of mmu_circ_0000349/mmu-miR-138-5p/JMJD3 axis on RUNX2.

## 5 Conclusions

**Fig. 8:**
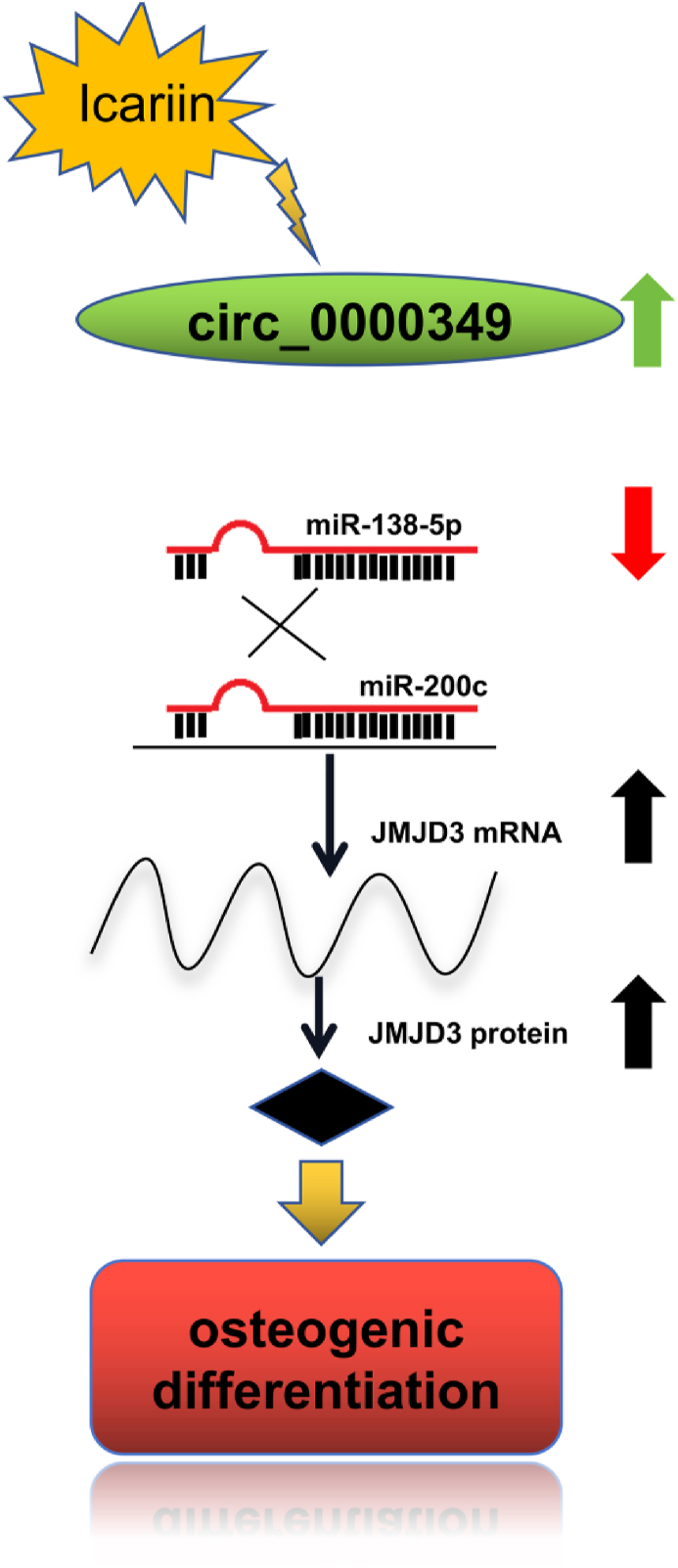
A mechanistic diagram of mmu_circ_0000349/mmu-miR-138-5p/JMJD3 axis regulated by icariin during the process of osteogenic differentiation.

In this study, JMJD3 expression was promoted in the progress of MC3T3-E1 osteogenic differentiation. Overexpressed mmu_circ_0000349 induced osteogenic differentiation; contrarily, decreased mmu_circ_0000349 exerted an opposite effect. mmu_circ_0000349 acted as a sponge of mmu-miR-138-5p to regulate JMJD3 (Fig. 8). Furthermore, with the increase in icariin concentration and time for treatment, osteogenic differentiation was induced by the regulation of the mmu_circ_0000349/mmu-miR-138-5p/JMJD3 axis.

## Data availability

All data generated or analyzed during this study are included in this published article [and its supplementary information files].

## Funding

This work was supported by the [10.13039/501100001809National Natural Science Foundation of China] (grant numbers [81774339] and [82074462]), the [Major research project of 10.13039/501100010618Guangzhou University of Chinese Medicine] (grant number [XK2019012]).

## CRediT authorship contribution statement

**Liang Ai:** Conceptualization, Data curation, Formal analysis, Resources, Writing – original draft. **Liudan Chen:** Data curation, Formal analysis, Methodology, Resources, Software, Writing – review & editing. **Yangu Tao:** Data curation, Formal analysis, Methodology, Resources, Writing – review & editing. **Haibin Wang:** Conceptualization, Data curation, Formal analysis, Methodology, Project administration, Resources, Software, Writing – review & editing. **Weimin Yi:** Data curation, Methodology, Resources, Software, Writing – review & editing.

## Declaration of competing interest

The authors declare that they have no known competing financial interests or personal relationships that could have appeared to influence the work reported in this paper.
